# What Do Customers Demand from Drug Stores in Japan? Construct Validity and Factor Structure of a Cross-Sectional Survey

**DOI:** 10.3390/pharmacy6030098

**Published:** 2018-09-06

**Authors:** Yuuki Minamida, Naoko Yoshida, Mio Nishimaki-Tomizu, Misato Hanada, Kazuko Kimura, Hirohito Tsuboi

**Affiliations:** 1 School of Pharmacy, Kanazawa University, Kanazawa 920-1192, Japan; minamida.yuuki@stu.kanazawa-u.ac.jp (Y.M.); dmpc14@p.kanazawa-u.ac.jp (M.N.-T.); dmpc12@p.kanazawa-u.ac.jp (M.H.); 2 Institute of Medical, Pharmaceutical and Health Sciences, Kanazawa University, Kanazawa 920-1192, Japan; naoko@p.kanazawa-u.ac.jp (N.Y.); kimurak@p.kanazawa-u.ac.jp (K.K.)

**Keywords:** drug store, customers demand, factor analysis, over-the-counter medicine, questionnaire

## Abstract

Studies concerning patient demands are mainly conducted at hospitals and pharmacies, whereas few surveys have been conducted on drug stores. The demand for drug stores is estimated to be increasing with growing needs for self-medication. Thus, conducting a customer survey at drug stores is thought to be valuable. The aim of the current study was to clarify the structure of customers’ demands for drug stores. The survey was conducted on 190 customers of 19 drug stores in Japan. The questionnaire consisted of 24 items using a 9-point Likert scale. The IBM SPSS Statistics version 23 (IBM Japan, Tokyo, Japan) and Amos version 5 (IBM Japan, Tokyo, Japan) were utilized to perform factor analysis. Gender did not influence the response to each question. Factor analysis showed that the structure of customers’ demands consisted of three factors: (1) an explanation about medicine, (2) staff’s manners, and (3) location of drug stores. Because fit indices suggested a good fit, this three-factor solution was adopted as the final factor structure. This study demonstrated the structure of customers’ demands for drug stores, with the potential for use in promotion of self-medication.

## 1. Introduction

Surveys on medical facilities were mainly conducted at hospitals and pharmacies [1,2,3,4,5,6,7,8,9,10,11]. However, there have been few reports about surveillance at drug stores. In Japan, prescription medicines are obtained from pharmacies, while over-the-counter (OTC) medicines may be obtained from a drug store. In addition, the law on tax deduction for self-medical expenses was enforced on 1 January 2017. Under the new law, many Japanese people are exempt from paying taxes on the purchase prices of switch OTC medicine. Switch OTC medicine is a kind of OTC medicine that contains the same active pharmaceutical ingredients as prescription drugs. Accordingly, the demand for OTC medicine is estimated to be increasing. Because OTC medicine is mainly sold at drug stores, the demand for the drug stores is also estimated to be increasing. Therefore, it is possible to say that conducting a customer survey at the drug store will be valuable.

A previous survey of patient expectations from pharmacies in Estonia indicated that more than half of the participants valued the pharmacy location when choosing a pharmacy, whereas one-fifth of them valued lower of pieces, and one-sixth valued a wide choice of products [9]. This survey also demonstrated that patients expected a professional consultation and help for choosing the right medicine the most [9]. An investigation on pharmacies in Japan clarified the structure of patient satisfaction: that is, (1) human factors, (2) patient’s convenience, and (3) environmental factors. Human factors included the pharmacists’ reception and explanation about medicines. The patient’s convenience factor included the pharmacy location and business hours. The environmental factors contained the environment of the waiting room and suitability of payment method [6]. These previous surveys revealed that patients at pharmacies considered the expertise and the pharmacy location as the most important factors.

Drug stores are slightly different from pharmacies in their function. Customers use drug stores to purchase OTC medicines or daily necessities, whereas patients use pharmacies for prescriptions. Because this difference may affect the structure of customers’ demands, it is worthwhile to investigate the customers of drug stores. The purpose of the current study was to clarify the structure of customers’ demands for drug stores.

## 2. Participants and Methods

### 2.1. Study Population and Procedure

A cross-sectional survey by convenience sampling was conducted on 190 customers of 19 stores across 5 prefectures (Ishikawa, Toyama Niigata, Fukui, and Nagano) belonging to a drug store chain, located close to researchers. Data were collected between 9 August and 9 November in 2012. Researchers placed paper questionnaires on the cashier’s table, and shop assistants or clerks asked customers to fill out the questionnaire. The answer sheets were collected on the spot or mailed to Kanazawa University. We gave a small gift (a dishwashing detergent or cling film) to each customer who answered the questionnaire. All customers were asked for their informed consent to participate. The proposal for this study was reviewed and approved by the Kanazawa University medical ethics committee (receipt number 34).

### 2.2. Questionnaire

The questionnaire consisted of two sections. The first section collected demographic details including age, gender, occupation, the frequency of using drug store, and medicines or supplements usage. The second section consisted of 24 items including customers’ demands for a positive staff manner, staff’s response, an explanation of medicines, characteristics of stores, and convenience. A nine-point Likert scale was used (Table 1), from 1 “unnecessary” through 5 “don’t know” to 9 “necessary”.

### 2.3. Analysis

Data were analyzed using the Japanese version of SPSS Statistics version 23 (IBM Japan, Tokyo, Japan) and Amos version 5 (IBM Japan, Tokyo, Japan). Participants with missing data and participants who checked the same scale number more than 90% were excluded from the analysis. The nine-point Likert scale answer was converted into a four-point scale to bring the data closer to a normal distribution: 9 converted to 4, 6–8 to 3, 5 to 2, and 1–4 to 1). Customers’ characteristics were compared using chi-square tests. *P*-values less than 0.05 were considered to be statistically significant. To clarify the structure of customers’ demands for drug stores, an exploratory factor analysis (EFA) and a confirmatory factor analysis (CFA) were calculated.

EFA was performed using maximum likelihood estimation and promax rotation. Kaisers criterion and scree plot were used as criteria for factor extraction. In order to make factor easy to understand, a factor loading greater than 0.60 was considered significant for retention. The reliability of each component was assessed using Cronbach’s alpha, for which a value above 0.80 was used as the criterion for adequacy.

CFA was performed to evaluate the construct validity. Maximum likelihood estimation was performed to calculate item loading. Items with factor loading less than 0.60 were removed from the model. To evaluate the goodness of fit of the model, chi-square, goodness of fit index (GFI), adjusted goodness of fit index (AGFI), comparative fit index (CFI), and root mean square error of approximation (RMSEA) were examined. In the present survey, the criterion for adequacy of each fit index was GFI > 0.90, AGFI > 0.90, CFI > 0.95, and RMSEA < 0.10 [12].

## 3. Results

### 3.1. Participants’ Demographics

A total of 190 customers (36 men and 154 women with mean age of 52.1 ± 17.56 and 16.2 ± 15.63, respectively) responded to the survey, and 26 of the questionnaires were excluded due to the criteria mentioned in analysis section. The number of responses and response rate were 164 (129 men, 35 women) and 86.8%, respectively. The number of answers from females were significantly higher than that from males (*t* test, *p* < 0.001). However, there were not significant differences between men and women concerning answers (*t* test).

### 3.2. Exploratory Factor Analysis

Due to the low factor loading, 12 items were removed, and a three-factor solution was adopted. This solution explained 70.2% of the variance. Table 2 shows the pattern matrix. No factor was cross-loaded. The three factors were labelled as (1) an explanation of medicine, (2) staff’s manner, and (3) location of drug stores. Analysis of reliability of each component presented good internal correlation with Cronbach’s alpha of 0.94 for an explanation of medicine and 0.88 for staff’s manners, whereas the Cronbach’s alpha of 0.79 for location was nearly good.

### 3.3. Confirmatory Factor Analysis

**Figure 1:**
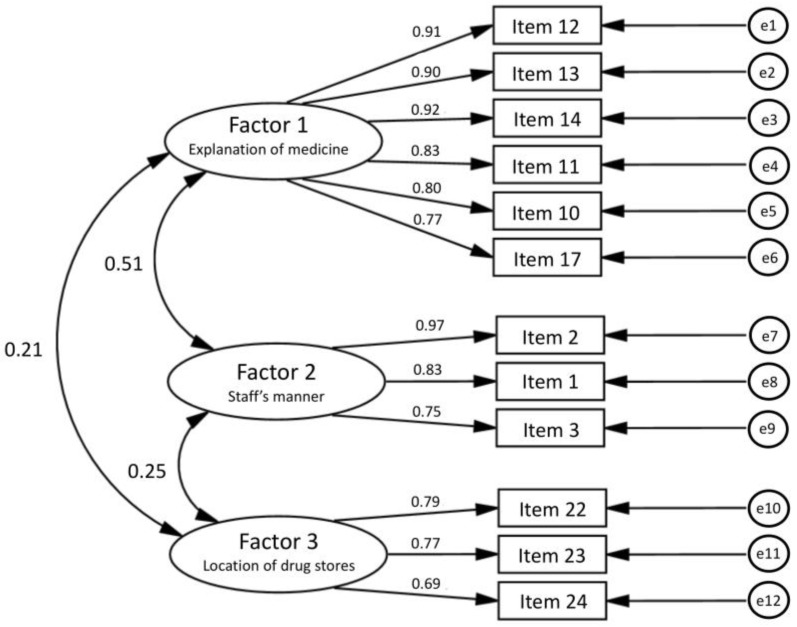
Standardized regression weights of the confirmatory factor analysis. Factor 1 explains medicine; Factor 2 staff’s manner; and Factor 3 location of drug stores. The content of each item is presented in Table 1. The labels from e1 to e12 shows the error included in each answer. The numbers near the unidirectional arrows are the standardized path coefficients. Correlation coefficients between factors are depicted next to bidirectional arrows.

After mapping the responses to the suggested model determined by the EFA, no item was removed, and the final factor structure was adopted (Figure 1). The chi-square value for overall model fit was chi-square (51) = 117.2, *p* < 0.001. Other fit indexes were GFI = 0.904, AGFI = 0.854, CFI = 0.953, and RMSEA = 0.089. The chi-square value suggested a significant misfit between the data and the model. However, it is known that in larger scale analyses, the chi-square value can be over-sensitive. Thus, other fit indexes are applied to decide whether the model is a good fit or not [12]. Because other fit indexes with the exception of AGFI suggested a good fit for the model and these three factors were easy to understand, this three-factor solution was determined as the final factor structure.

## 4. Discussion

This survey has clarified the structure of customers’ demands for drug stores in Japan. The results of EFA and CFA showed that the structure of customers’ demands consisted of three factors: (1) an explanation of medicine, (2) staff’s manners, and (3) location of drug stores. This result indicated that customers requested service and convenience more than the medicine itself.

In 2015, the Ministry of Economy, Trade and Industry (METI) of Japan held a study to promote self-medication and issued a report [13]. The METI report presented that customers evaluated a drug store’s location and convenience highly, and expertise of the drug store was not valued. This result was different from the present survey. In the METI report, customers did not value a drug store’s expertise, which includes staff’s knowledge. However, in our survey, expertise of the drug store was valued. There were several possible reasons why such a difference occurred. First, the location of participants was different. Participants in the METI survey were females in the Tokyo area. In the present survey, we investigated residents living in suburbs and rural areas in the central part of Japan. Second, analysis methods of the two researches were different. The METI report presented just the proportion of customers’ demands. In our survey, a robust two-staged analytical method, involving EFA and CFA, was used to assess the structure of customers’ demands. Finally, the way of collecting answers was different. The METI research was conducted on the internet, whereas we collected answers at the drug stores using paper questionnaires. Those differences such as location and survey methods could have affected the participants’ demographics and influenced the results but could also have resulted from the different analysis methods.

### 4.1. Participants’ Demographics

In this research, the number of female responders was significantly higher than that of male responders. This indicated that most of the drug store’s customers were women, because we took questionnaires at random. Most drug stores deal in food, daily necessities, and cosmetics. These goods are mainly in demand by females, and this may affect the proportion of responder’s gender.

### 4.2. Exploratory Factor Analysis

Cronbach’s alpha for location was 0.79, which was nearly a good result. Cronbach’s alpha is considered as a reliability coefficient when the following conditions are met: (1) there is no correlation between errors, (2) the whole scale has a one-factor solution, and (3) path coefficients from one factor to all observed variables are equal [14]. When the conditions are not met, it is known that Cronbach’s alpha becomes smaller than reliability coefficient. In this survey, path coefficients from one factor to all observed variables were not equal; thus, the conditions were not met and the Cronbach’s alpha of 0.79 for location was smaller than the reliability coefficient. Therefore, the Cronbach’s alpha of 0.79 for factor (3) location of drug stores can be said to fall in the range of acceptable internal consistency.

### 4.3. Confirmatory Factor Analysis

The final factor structure consisted of three factors: (1) an explanation of medicine, (2) staff’s manner, and (3) location of drug stores. As mentioned above, the previous studies at pharmacies demonstrated that patients visiting pharmacies considered the expertise and the pharmacy’s location as the most important [2,6,7,9,10,11]. The current study revealed that the functional difference between drug stores and pharmacies had some effects on the structure of customers’ demands. This result indicated that customer demands for OTC medicines were almost equal to prescription medicines and that professional advice was necessary to use both medicines. On the other hand, many customers demanded convenient location of drug stores and pharmacies. Stores with a convenient location could have an advantage over other stores.

### 4.4. Limitations

Because customers were selected from a chain of drug stores in some parts of Japan in the present survey, there might be several types of selection bias based on demographics and characteristics of the participants in different regions or countries. Future studies are needed on residents in other regions with larger population.

## 5. Conclusions

We clarified the structure of customers’ demands for drug stores: (1) explanation of medicine, (2) staff’s manner, and (3) location of drug stores. To our knowledge, this is the first report showing these factors in Japan. Given that there is growing recognition of the need for self-medication, our results are meaningful to drug stores for improving customer satisfaction, which affects the smooth spread of self-medication.

## Figures and Tables

**Table 1:** Questionnaire.

No.	Items
1	Staff pay attention to their appearance.
2	Staff use appropriate language.
3	Staff have a very expressive face.
4	Staff talk to customers or make a greeting
5	Staff are privacy-conscious.
6	Length of medical explanation is appropriate.
7	Staff answer my questions or worries.
8	Is easy to consult staff.
9	Staff take good care of me.
10	Staff explain the effect of medicine.
11	Staff explain the direction and dosage of medicine.
12	Staff explain the side effects of medicine.
13	Staff explain the drug interaction of medicine.
14	Staff explain the cautions needed when taking medicine.
15	Staff explain the difference between the medicine and a similar drug.
16	Staff explain the price of medicine.
17	Staff explain how to take care of yourself in everyday life.
18	Store contains many products.
19	It is easy to understand the placement of medicine.
20	Store is clean and hygienic.
21	Medicine is inexpensive.
22	Distance and the time from home to the store.
23	Convenience of traffic from home to the store.
24	Business days and business hours are convenient.

Participants responded to each of these 24 items using a 9-point Likert scale.

**Table 2:** EFA rotated factor structure.

Item No.	Components		
	1	2	3
	α = 0.94	α = 0.88	α = 0.79
12	0.94		
13	0.90		
14	0.88		
11	0.85		
10	0.79		
17	0.77		
2		1.02	
1		0.77	
3		0.75	
22			0.86
23			0.76
24			0.61

EFA: exploratory factor analysis.

## References

[1] Solheim A.M., Mygland Å., Ljøstad U. (2017). Quality of multiple sclerosis out-patient health care services with focus on patient reported experiences. BMC Res. Notes.

[2] Chan V., Tran H. (2016). Purchasing over-the-counter medicines from Australian pharmacy: What do the pharmacy customers value and expect?. Pharm. Prac. (Granada).

[3] Yang S., Kim D., Choi H.J., Chang M.J. (2016). A comparison of patients’ and pharmacists’ satisfaction with medication counseling provided by community pharmacies: A cross-sectional survey. BMC Health Serv. Res..

[4] Malewski D.F., Aimrie R., Caroline A.G. (2015). Patient satisfaction with community pharmacy: comparing urban and suburban chain-pharmacy populations. Res. Soc. Adm. Pharm..

[5] Sakurai H., Konno H., Shimamori Y., Sugiyama H., Yoshimachi M., Kouno H., Gotou T., Hayase Y. (2009). Investigation on patient’s and pharmacist’s attitudes toward medical services in community pharmacies. J. Pharm. Soc. Jpn..

[6] Sakurai H., Nakajima H., Tada Y., Yoshikawa E., Iwahashi Y., Fujita K., Hayase Y. (2009). An investigation on pharmacy functions and services affecting satisfaction of patients with prescriptions in community pharmacies. J. Pharm. Soc. Jpn..

[7] Chen P.T. (2007). A survey of factors influencing customer satisfaction at dispensing pharmacies. Shinshu Med. J..

[8] Sakurai H., Kawahara S., Tada Y., Nakajima F., Igari T., Momose H., Kondo H., Komori Y., Hayase Y. (2007). Investigation on patient satisfaction at community pharmacies: analyzing questionnaire survey by structural equation modeling and multiple regression analysis. J. Pharm. Soc. Jpn..

[9] Villako P., Raal A. (2007). A survey of Estonian consumer expectations from the pharmacy service and a comparison with the opinions of pharmacists. Pharm. World. Sci..

[10] Hayashi S., Hayase T., Mochizuki M., Hashiguchi M., Takeuchi K. (2005). Classification of pharmaceutical services from the viewpoint of patient satisfaction/dissatisfaction. J. Pharm. Soc. Jpn..

[11] Kamei M., Teshima K., Fukushima N., Nakamura T. (2001). Investigation of patients’ demand for community pharmacies: Relationship between pharmacy services and patient satisfaction. J. Pharm. Soc. Jpn..

[12] Komatsu M., Toyoda H. (2007). Chapter 1—Introduction to AMOS. Covariance Structure Analysis.

[13] The Ministry of Economy, Trade and Industry (METI) of Japan (2015). A Report for the Role of Drug Stores in Relation to Promotion of Self-Medication. http://www.meti.go.jp/press/2014/03/20150313004/20150313004a.pdf.

[14] Murohashi H., Toyoda H. (1998). Chapter 3—Regression Analysis. Covariance Structure Analysis.

